# Identification of Potential Plk1 Targets in a Cell-Cycle Specific Proteome through Structural Dynamics of Kinase and Polo Box-Mediated Interactions

**DOI:** 10.1371/journal.pone.0070843

**Published:** 2013-08-15

**Authors:** Nousheen Bibi, Zahida Parveen, Sajid Rashid

**Affiliations:** National Center for Bioinformatics, Quaid-i-Azam University, Islamabad, Pakistan; National Cancer Institute, NIH, United States of America

## Abstract

Polo like kinase 1 (Plk1) is a key player in orchestrating the wide variety of cell-cycle events ranging from centrosome maturation, mitotic entry, checkpoint recovery, transcriptional control, spindle assembly, mitotic progression, cytokinesis and DNA damage checkpoints recovery. Due to its versatile nature, Plk1 is considered an imperative regulator to tightly control the diverse aspects of the cell cycle network. Interactions among Plk1 polo box domain (PBD) and its putative binding proteins are crucial for the activation of Plk1 kinase domain (KD). To date, only a few substrate candidates have been characterized through the inclusion of both polo box and kinase domain-mediated interactions. Thus it became compelling to explore precise and specific Plk1 substrates through reassessment and extension of the structure-function paradigm. To narrow this apparently wide gap in knowledge, here we employed a thorough sequence search of Plk1 phosphorylation signature containing proteins and explored their structure-based features like conceptual PBD-binding capabilities and subsequent recruitment of KD directed phosphorylation to dissect novel targets of Plk1. Collectively, we identified 4,521 phosphodependent proteins sharing similarity to the consensus phosphorylation and PBD recognition motifs. Subsequent application of filters including similarity index, Gene Ontology enrichment and protein localization resulted in stringent pre-filtering of irrelevant candidates and isolated unique targets with well-defined roles in cell-cycle machinery and carcinogenesis. These candidates were further refined structurally using molecular docking and dynamic simulation assays. Overall, our screening approach enables the identification of several undefined cell-cycle associated functions of Plk1 by uncovering novel phosphorylation targets.

## Introduction

During mitosis, faithful distribution of chromosomes to newly forming daughter cells necessitates careful coordination of multiple processes such as entry into mitosis, spindle assembly, chromosome segregation and cytokinesis. Polo like kinases (Plks) are emerging as key regulators of essential cell cycle events and have garnered a lot of attention [1], [2], [3].

Plks belong to serine/threonine kinase family, originally identified from a mitotic mutant of *Drosophila melanogaster* and was named “POLO” due to the presence of abnormal spindle poles [4], [5]. Plks are highly conserved from baker's yeasts to humans with specific and dynamic roles throughout cell-cycle process [6], [7], [8], [9]. Plk1 localizes to the cytoplasm and centrosome during interphase and concentrates to kinetochores and the cytokinesis bridge during cell division. Thus this protein has major functions in centrosome maturation, mitotic entry, and cytokinesis [10], [11]. Plk1 has also emerged as a novel modulator of DNA damage checkpoints, where it maintains genomic stability during DNA replication [11].

In mammals, the Plk family is comprised of four members (Plk1, Plk2/Snk, Plk3/Prk/Fnk and Plk4/Sak1) [12]. All Plks contain an N-terminal Ser/Thr kinase catalytic domain and a C-terminal region containing two conserved POLO-Box regions (PBDI; 411–489 AA and PBDII; 511–592 AA); however, they share only 12% sequence identity [10]. Polo box domain (PBD) of Plk1 plays a unique role in subcellular localization and recruitment of substrates through coordinating the binding affinity and specificity of kinase-substrate interactions [13], [14]. The analysis of Plk1 KD and PBD has suggested that these domains contribute in transient interactions intramolecularly and inhibit each other. Specialized recognition of PBD to ligand(s) in a phosphorylation-dependent manner releases the KD and relieves this inhibitory interaction [15], [16], [17]. Hence PBD serves as an essential molecular mediator to regulate localization and substrate selectivity in time and space.

Despite the significant and pleiotropic functions of Plk1 in coordinating cell-cycle progression, surprisingly only few specific targets are known which are uniquely phosphorylated by Plks. In order to explore Plk1-related cascade of molecular events, understanding of possible links between potential phosphorylation sites and subsequent interacting proteins is crucial [10], [18], [19]. Thus, linking a certain kinase with particular phosphorylation events remains intricate and elucidation of kinase-substrate relationships is vital in understanding the intracellular signal transduction and cellular physiology.

Even though the well known contributions of Plk1 in mitotic processes and extensive identification of mitotic targets including Cdc25C [20], Brca2 [21], Nlp [22], TCTP [23], MKlp1 [24], [25], Myt1 [26], Cyclin B [20], [27], NudC [28], MKlp2 [17], Grasp65 [29], [30] and Wee1 [31], [32], only a few of them contain specific PBD binding and kinase phosphorylation motifs for Plk1. For example, Cdc25C, Brca2, MKlp2, Grasp65, Wee1, and Myt1 are also phosphorylation targets of Cdk1 [16]. Similarly, NudC, cyclin B, and TCTP are phosphorylated by mitotic kinases other than Plk1 [13] and many substrates identified through tracking of PBD-binding sites lack putative phosphorylation motifs [33]. These data suggest that knowledge of Plk1 targets based on the consensus site information for the PBD recognition and kinase specific phosphorylation motifs is incomplete. Moreover, only a small number of substrate candidates have been structurally validated so far [9]. Hence, mapping of targets in terms of their involvement in precise cell-cycle phase remains important to address their unique roles in cellular events.

Here, in an effort to identify Plk1-based novel PTM targets involved in cancer phenotype and cell-cycle progression, we have merged *in silico* sequence and structure-based approaches. Our findings are intended for pathway elucidation by focusing the underlying kinase-substrate relationships leading to cancer diagnosis and effective target-directed therapeutics.

## Methods

### Identification of putative phosphorylation targets of Plk1

Compilation of phosphorylation site consensus motif is a contemporary strategy to predict novel PTMs target of a particular kinase. Target proteins that hold an instance of a given kinase specific consensus motif are predicted to be substrates of that kinase. In this context linear motif searching is the most simple and currently a widely used approach. In an attempt to identify novel substrates for Plk1, initially we assessed the adequacy of consensus phosphorylation motif **[E/D]X[pS/pT][I/L/V/M]X[E]** for Plk1 KD [26], which was shared by a majority of Plk1 substrates. Sequence based computational tool such as BLAST (Basic Local Alignment Search Tool) [34] was employed to search the short peptide motifs against non redundant set of known human proteins (All non-redundant GenBank CDS translations +PDB +Swissprot +PIR +PRF) with the following parameters: number of sequences: 23618720, expected threshold value: 10, word size: 3, scoring matrix: BLOSUM62, gap existence penalty: 11 and gap extension penalty: 1. Identified hits were ranked according to the similarity with original motif.

### Screening of Plk1 specific substrates

Series of filters were applied to these hits in order to search for Plk1 candidate substrates and to avoid the chance of false positive results. The set of filters integrated: (i) incidence of Plk1 phosphorylation motif. (ii) 80% identity with the **[E/D]X[pS/pT][I/L/V/M]X[E]** consensus phosphorylation motif: putative substrate hits exhibiting more than 80% identity were selected. (iii) Gene Ontology (GO) [35]: proteins were clustered on the basis of their involvements in common biological processes, molecular functions and cellular component localization. Subsequently, statistical significance of hit-rate was computed as the probability *P* to observe the by chance occurrence of each cluster.



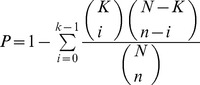
Where, (*N*) is the total number of annotated proteins in a non redundant human protein data set (NCBI nr), (n) denotes the number of annotated proteins in a given cluster, (*k*) is the number of proteins out of *n* proteins in the cluster, annotated on the basis of gene ontology (GO), (*K*) represents the number of proteins out of *N* proteins, annotated for the function, complex or pathway of interest [33], [36]. All enrichments with *p*-values >0.1 were discarded from further analysis. (iv) Resulting hits were evaluated for priming phosphorylation and PBD binding capabilities on the basis of presence of Plk1 PBD recognition consensus motif [**S-(pT/pS)-(P/X)**]. (v) Selected protein clusters were then filtered out on the basis of their functional contributions in cell-cycle process and carcinogenesis. Individual cell cycle phase specific contributions of candidate proteins were determined using functional annotation (GO term, Reactome Plugin of Cytoscape 2.8.3 and KEGG pathway enrichment) and cluster analysis. For cancer specific roles, Reactome Functional Interaction cancer gene index was loaded to identify the putative cancer associated clusters from the candidate clusters of cell cycle. Protein interaction network mapping analysis and visualization were performed using Cytoscape 2.8.3 (http://www.cytoscape.org). Subsequently, Netphos 2.0 [37], GPS [38], phospho.ELM database [39] and HPRD (http://www.hprd.org/PhosphoMotif_finder) were used to compare and verify the predicted phosphorylation and binding sites in the selected targets.

### 3D structure modeling of putative Plk1 substrates and KD

Putative substrates isolated by the above-mentioned filters were selected for protein modeling analysis. Homology modeling and *ab initio* structure prediction approaches were employed to predict the models for candidate proteins (in case of proteins lacking experimentally determined PDB structures). Primary protein sequences for 18 selected (both known and novel) substrate candidates (encompassing Plk1-PBD recognition and kinase phosphorylation motifs) and Plk1-KD were retrieved through Ensemble genome browser [40] and subjected to BLAST search against PDB (Protein Data Bank) database for suitable template search. 3D structures for the candidate proteins having good template (100–150 aa encompassing Plk1 kinase phosphorylation and PBD binding motif) were determined by Modeller9V8 tool [41], using homology modeling approach. For proteins lacking a good template, 3D structures were predicted through I-TASSER [42] and Muster [43] servers, using *ab-initio* approach. Subsequently, Ramachandran plot, PROCHECK, ERRAT [44], VERIFY 3D [45], and WHAT IF [46] tools were utilized to validate the predicted 3D models, followed by model refinements, editing (phosphorylation) and geometry optimizations using WinCoot [47], UCSF Chimera 1.7.0 [48] and VEGA ZZ (http://www.ddl.unimi.it) tools.

### Molecular docking analysis

Modeled peptides for highly ranked hits (putative novel and three known Plk1 substrates including CEP10, KIF23 and BRCA2) were docked against polo box and kinase domains of Plk1 using automated molecular docking tool AutoDock 4.0 [49], HADDOCK [50] and flexible protein-protein docking server named as SwarmDock [51]. 3D structures of Plk1-KD and PBD (PDB ID: 1Q4K) were used as the receptors for performing dockings against the 13 chosen substrate hits (SMARCAD1, ZNF791, GSG2, NUP35, NEK5, MARK1, HUS1B, SIK2, TOP3B, MARK4, KIF23, CEP170 and BRCA2). Briefly, polar hydrogen atoms were added and kollman charges were assigned for ligands and all torsions were set free to rotate in order to perform docking experiments with a rigid receptor and flexible ligands. Annealing parameters for hydrogen bonding and Van der Waals interactions were set to 4.0 Å and 2.5 Å. A grid map of 80×80×80 points with a spacing of 0.875 Å was set on the whole protein structure to generate the grid map. The number of runs for each docking experiment was set to 100. The empirical free energy function and Lamarckian genetic algorithm (LGA) were applied with the following parameters: population of 150 randomly placed individuals, a maximum number of 27,000 generations, a mutation rate of 0.02, a crossover rate of 0.80 and number of energy evaluations was 2.5×10^6^, the remaining docking parameters were set to default. The program automatically grouped potential receptor-ligand complex conformations into clusters based on their RMSD, using the default threshold (2.0 Å RMSD). The best docked complex for each ligand was selected and interactions were monitored using DIMPLOT [52].

### Molecular dynamic simulations

Molecular dynamic (MD) simulations of the four best docked Plk1-substrate complexes (Plk1-SMARCAD1, Plk1-NEK5, Plk1-NUP35, and Plk1-GSG2) were performed to evaluate the stability, folding, conformational changes and dynamic behavior of interacting proteins. All MD simulations were performed using Amber03 force field using GROMACS 4.5 package [53], running on high performance OpenSuse linux system. During MD simulations, all the systems were solvated using TIP4P [54] water model in a periodic box, followed by addition of Na^+^ and Cl^−^ counter ions to neutralize the systems. Before MD simulations, energy minimization (steepest descent algorithm for 500 steps) was performed by tolerance of 1000 kJ/mol Å^2^ to remove initial steric clashes. The energy minimized systems were treated for 1000 ps equilibration run under pressure and temperature conditions to relax the systems. Finally, MD simulations were run for 20 ns time scale under constant temperature (300 K) and pressure (1 atm). PME (Particle Mesh Ewald) algorithm was used in all calculations to dissect electrostatic interactions. Snapshots were collected throughout the MD simulations of each system and PDBs were generated for 5, 10, 15 and 20 ns intervals to investigate the time-dependent behavior and stability of each system. VMD [55], PyMol (http://www.pymol.org) and GROMACS tools were used to analyze the stability and behavior of each system.

## Results

### Identification of novel Plk1 specific substrates

**Figure 1:**
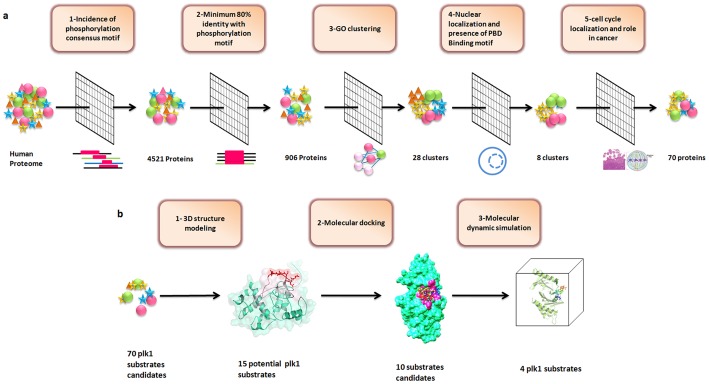
Schematic illustration of strategy. (a) Plk1 candidates substrates were selected on the basis of series of filters applied to human proteome including, presence of Plk1 specific phosphorylation motif at sequence level, minimum 80% identity, clustering on the basis of biological process, cellular localization and molecular function, presence of PBD recognition motif, common cell-cycle localization and role in cancer. (b) Scheme depicting different structure-based techniques used for the validation of putative hits obtained from sequence-based approaches.

In order to identify novel substrate candidates for Plk1, we developed a filtering approach to increase the specificity of our predictions by integrating multiple types of relative data sets including percent identity, clustering analysis GO (Gene Ontology) database, subcellular localization, presence of kinase phosphorylation and PBD recognition motif and role in cancer (Figure 1). We focused our analysis on cell-cycle proteins playing unique roles in distinct cell-cycle phases with well-defined contributions in multiple cancer types.

**Figure 2:**
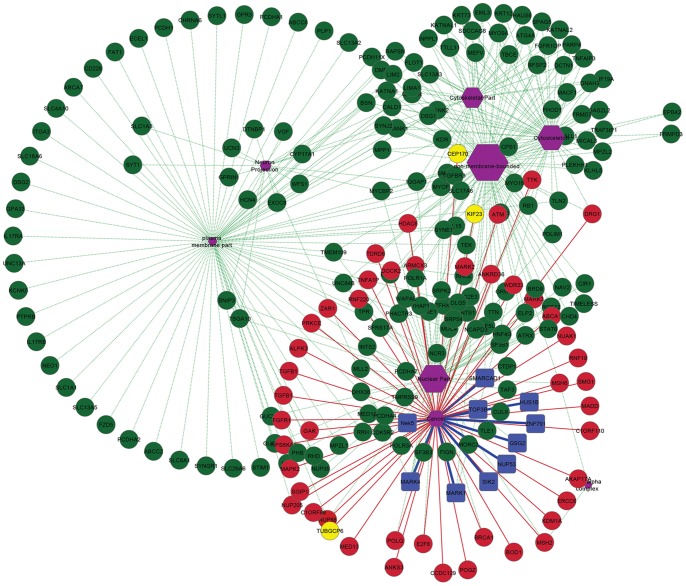
Gene network clusters enriched in cancer and cell-cycle related nodes. Red nodes represent proteins involved in cancer and cell-cycle, yellow are known Plk1 substrates, while green nodes represent targets having no known contributions in cancer. The blue nodes are proteins selected for structural validation in this study. Green and red lines represent protein network edges.

Protein Basic Local Alignment Search Tool (BLAST) was used to determine the Plk1kinase specific consensus motif **([E/D]X[pS/pT][I/L/V/M]X[E])** against the non redundant set of human proteome. In total, 4,521 protein hits were attained as a result of local alignment of residues adjacent to the consensus motif phosphorylation site. At this level, filter was applied to restrict our analysis to the candidates having greater than 80% similarity to query consensus motif. By this threshold, the number of potential candidates was reduced to 906. These 906 candidate hits were further categorized on the basis of their involvement in biological process, cellular location and molecular function by GO term and resulting in 28 clusters (Table S1). To get rid of redundancy, clusters were carefully investigated and refined on the basis of statistical significance and hit rate (*ρ-*value) analysis [35], [36]. The resulting protein clusters (482 proteins) were comprehensively explored for the presence of an optimal PBD recognition motif **S-[pS/pT]-[P/X]** [14]. This motif was found amongst 48.7% of the short listed proteins. In total, we identified 8 clusters comprising 235 well known and novel targets of Plk1 (Figure 2). By increasing the specificity directed analyses, we confined our study to cell cycle proteome and focused only on proteins which retain extensive roles in cell-cycle process and cancer.

**Figure 3:**
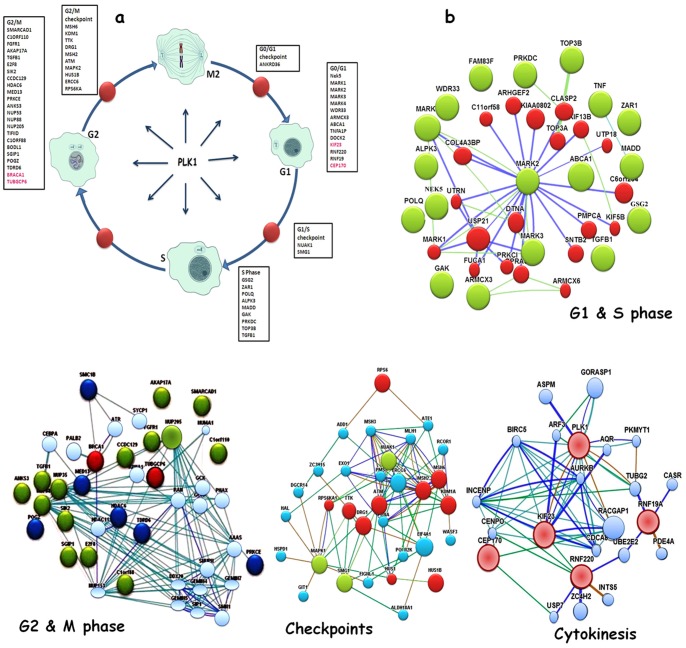
Plk1-dependent substrates. (a) Cell-cycle diagram depicting proteins sorted out through sequence based approach having Plk1 phosphorylation motif. Proteins are classified according to function and localization. Known Plk1 substrates are shown in red and novel putative substrates are shown in black. Red nodes indicate cell-cycle checkpoints. (b) Protein interaction analysis and interaction map in cell-cycle localized clusters (G1 and S, G2 and M, checkpoints, cytokinesis). Node color indicates the specific localization and other distinguishing features. In G1 and S phases, green nodes are G1 specific proteins and red nodes represent proteins involved in both G1 and S. In G2 and M phases, green nodes are both G2 and M specific and predicted as novel substrates in our study, red nodes are known Plk1 substrates and dark blue nodes are substrates shared by other kinesis. In checkpoints interaction map, red nodes represent G2/M specific checkpoints and green nodes are G1/S checkpoints. In cytokinesis interaction map, pink nodes are known Plk1 substrates. Blues lines delineate direct interactions, green lines represents co-expression, brown lines delineate shared protein domain.

We intersected 8 clusters on the basis of overlapping subsets and short listed 70 functionally annotated hits meeting the stringent criteria as listed in Figure 1a. These reputed hits were categorized in cell-cycle phase-dependent manner regarding the particular biological milieu of Plk1. Using Cytoscape tool; subnetworks were constructed for G1, S, G2/M, cytokinesis and checkpoints with cancer association to investigate the additional functions of Plk1 (Figure 3). Subsequently, Human protein Atlas (http://www.proteinatlas.org), CanProVar (http://bioinfo.vanderbilt.edu/canprovar), CanSar (https://cansar.icr.ac.uk) and COSMIC (http://www.sanger.ac.uk/genetics/CGP/cosmic) resources were used to substantiate and cross check the functions of preferred candidate proteins in a range of cancer types.

**Figure 4:**
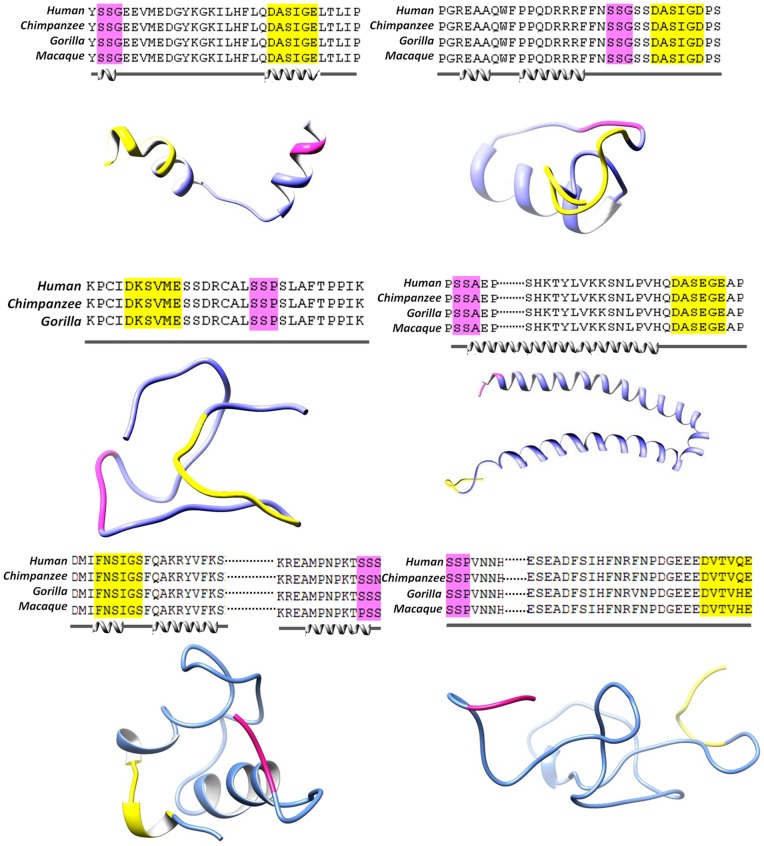
Multiple sequence alignment (MSA), secondary and tertiary structures of four selected Plk1 specific substrates. (a) SMARCAD1, (b) GSG2, (c) NUP35, (d) NEK5, (e) KIF23 and (f) CEP170. MSA illustrates the conservation pattern of predicted PBD recognition and kinase phosphorylation motifs across close homologues of selected proteins (a–d). Secondary structure for each substrate was predicted by JPred as shown below the alignments Tertiary structures of (a–f) substrates are shown in cornflower blue ribbon-form, phosphorylation motifs are shown in yellow, PBD recognition motifs are shown in pink.

Later, by using phosphorylation site prediction tools, including Netphos [37], GPS [38], phospho.ELM database [39] and HPRD (http://www.hprd.org/PhosphoMotif_finder), we further validated the prediction accuracy. Generally, evolutionary conserved sequences tend to be functionally important and to find these conserved elements, multiple sequence alignments (MSAs) (known and novel targets) were generated by ClustalW (Figure 4) [56].

### Structure-based approaches

To monitor conservation at secondary structure level, structure prediction was carried out by PSIpred server [57] (Figure 4). Secondary structure analysis for these substrates revealed the presence of abundant phosphorylation and PBD recognition motifs in the loop regions, which are generally easily accessible to the kinases for substrate binding and phosphorylation [58]. To substantiate our sequence-based screening results, candidate substrates including SMARCAD1, ZNF791, GSG2, NUP35, NEK5, MARK1, HUS1B, SIK2, TOP3B and MARK4 from each sub-network were randomly selected and subjected to multi-scale structure-based studies (Figure 1b). Additionally, three known Plk1 substrates KIF23 (also known as MKLP1) [25], CEP170 [59] and BRCA2 [21] were used for monitoring the accuracy of our strategy. 3D models of Plk1-KD and mentioned proteins were predicted using homology modeling and *ab-initio* structure prediction approaches. The models were refined and characterized by online structure analysis tools. Ramachandran plot indicated that approximately 95% residues of all the models lie in allowed regions. Moreover, parameters like peptide bond planarity, non-bonded interactions, Cα tetrahedral distortion, main chain H-bond energy and overall G factor for the modeled structures lie within favorable range. Errat measures the overall quality factor for non-bonded atomic interactions and an accepted range of above 50 is considered for a high quality model. In our case, Errat (ver. 2) scores were within the range of 82–95% (Table S2). These data indicated that our predicted structures are of good quality and can be used for structure-activity studies. Subsequently, their phosphorylation and PBD recognition signatures were estimated at structure level as shown for the representative candidate subsets (Figure 4: (a) SMARCAD1, (b) GSG2, (c) NUP35, (d) NEK5, (e) KIF23 and (f) CEP170). Phosphopeptides were generated by adding a phosphate group to respective residue of binding motif through UCSF Chimera 1.7.0 followed by energy minimization through VEGA ZZ tool using genetic algorithm. The coordinate files for the predicted structures of Plk1-KD and its substrate candidates have been submitted in Protein Model Database (http://mi.caspur.it/PMDB/) with following IDs: PM0079081 (human Plk1-KD); PM0078966 (SMARCAD1); PM0078956 (NUP35); PM0078970 (GSG2); PM0078958 (NEK5); PM0078969 (CEP170) and PM0078965 (KIF23).

### Phosphopeptide binding and conformational alterations

Plk1 binds to the peptide molecules phosphorylated (by priming kinases) at PBD recognition motif and consequently releases the KD for substrate phosphorylation [14]. In order to gain a deep insight into the mechanism of substrate phosphorylation by Plk1, 13 selected phosphopeptides (SMARCAD1, ZNF791, GSG2, NUP35, NEK5, MARK1, HUS1B, SIK2, TOP3B, MARK4, CEP10, KIF23 and BRCA2) were subjected to molecular docking simulations to monitor Plk1-substrate interactions.

**Figure 5:**
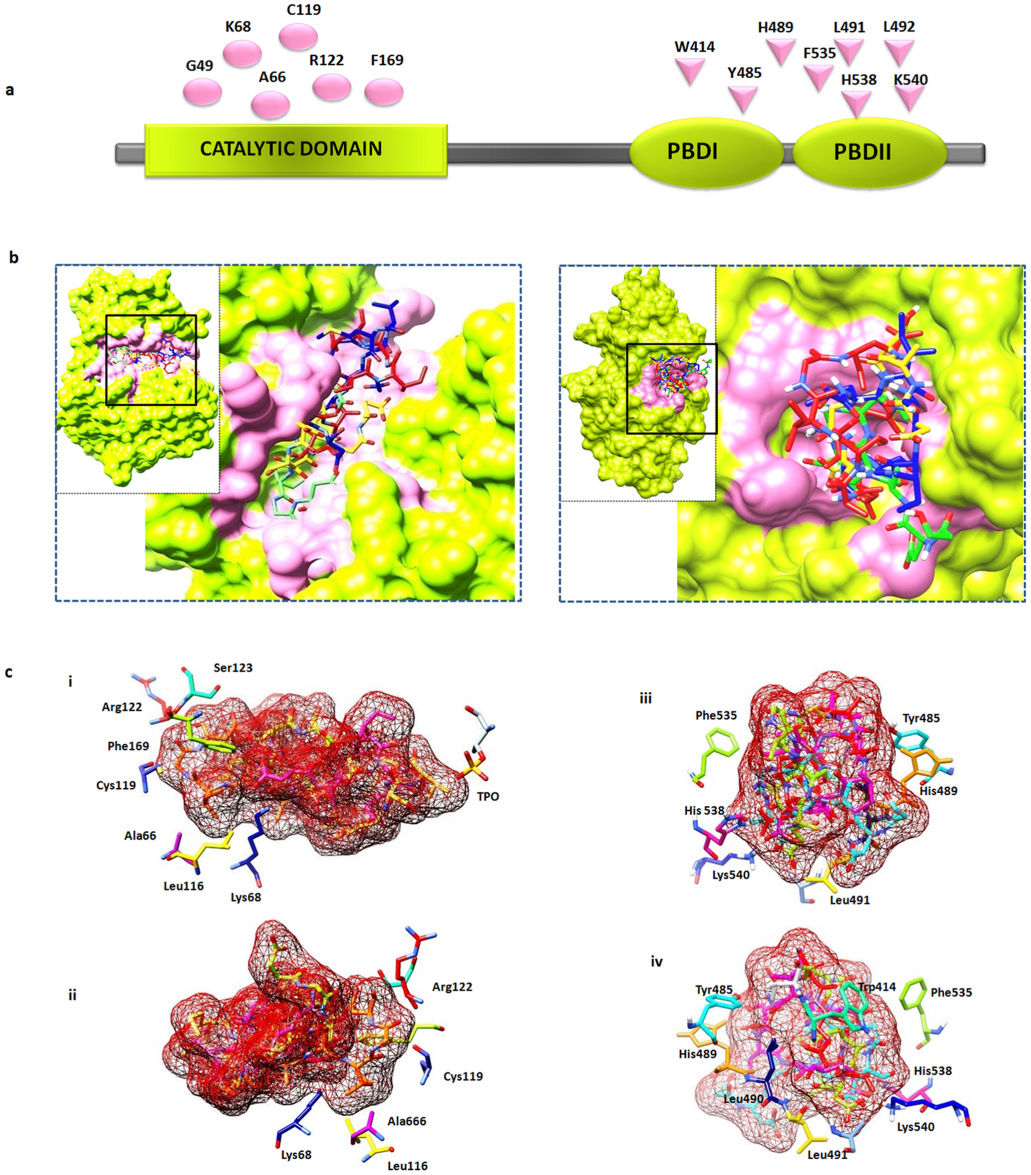
Plk1 protein architecture and substrates placing in the binding pockets of PBD and KD. (a) Schematic representation of Plk1 3D architecture: important residues lying at the KD and PBD (Polo Box domain involved in binding is highlighted by pink color); (b) Plk1 3D model, (i) KD, (ii) PBD with specific binding pocket (pink); selected Plk1 specific substrate candidates (putative novel and known) bind at the common position (with respect to motif signature) inside the binding pockets of KD and PBD. (c) Distinctive residues of Plk1 binding pocket and selected substrates; (i) KD anterior view and (ii) KD posterior view, (iii) PBD anterior view (iv) PBD posterior view.

Initially, solo domains of Plk1 were docked against the PBD recognition and kinase phosphorylation motifs to understand the specificity towards the respective motif and resultant conformational adjustments. We observed convergence of predominant docking clusters of phosphopeptides (both known and novel) at the substrate binding pocket of Plk1 PBD. Similarly, phosphorylation motif was found to be well placed in the binding pocket of Plk1 KD which clearly demonstrated their binding affinities and specificities for Plk1 polo box and kinase domains, respectively (Figure 5b–i and ii). Previously, it has been reported that Trp414, Tyr485, His489, His538 and Lys540 residues located in the periphery of Plk1 PBD are involved in substrate recognition and binding activities [14]. In our analysis, beside the contributions of these residues, Asp416, Leu490, Leu491 and Phe535 (located in the Plk1 binding pocket) were also observed in PBD association to phosphopeptides (Figure 5c–iii and iv). In contrast to PBD, Plk1 KD was observed to exhibit strong electrostatic interaction with the putative phosphorylation motif through Leu59, Gly63, Cys133, Arg135, Arg136, Ser137, Lys82, Lys86 and Phe183 residues (Figures 5c–i and ii).

**Figure 6:**
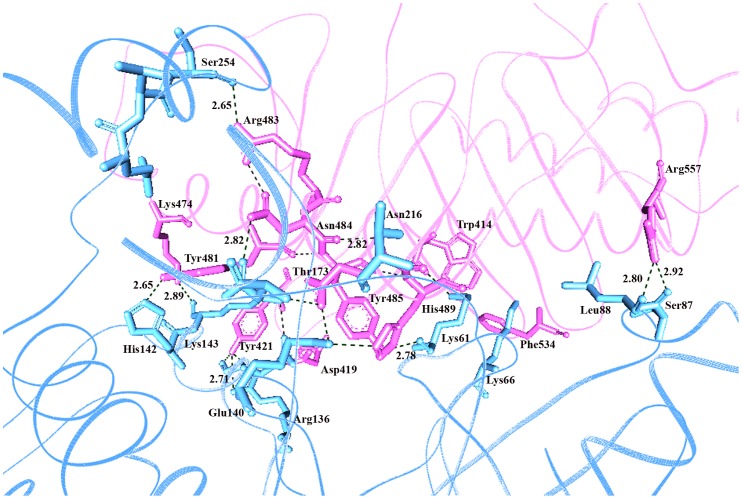
Characterization of inhibitory molecular interaction between PBD and KD of Plk1. Cyan blue meshy ribbon represents the KD with interacting residues in cyan stick form, while PBD is represented in pink meshy ribbon with interacting residues in pink stick. Hydrogen bonds are represented by black dotted lines with bond distances in angstrom.

**Figure 7:**
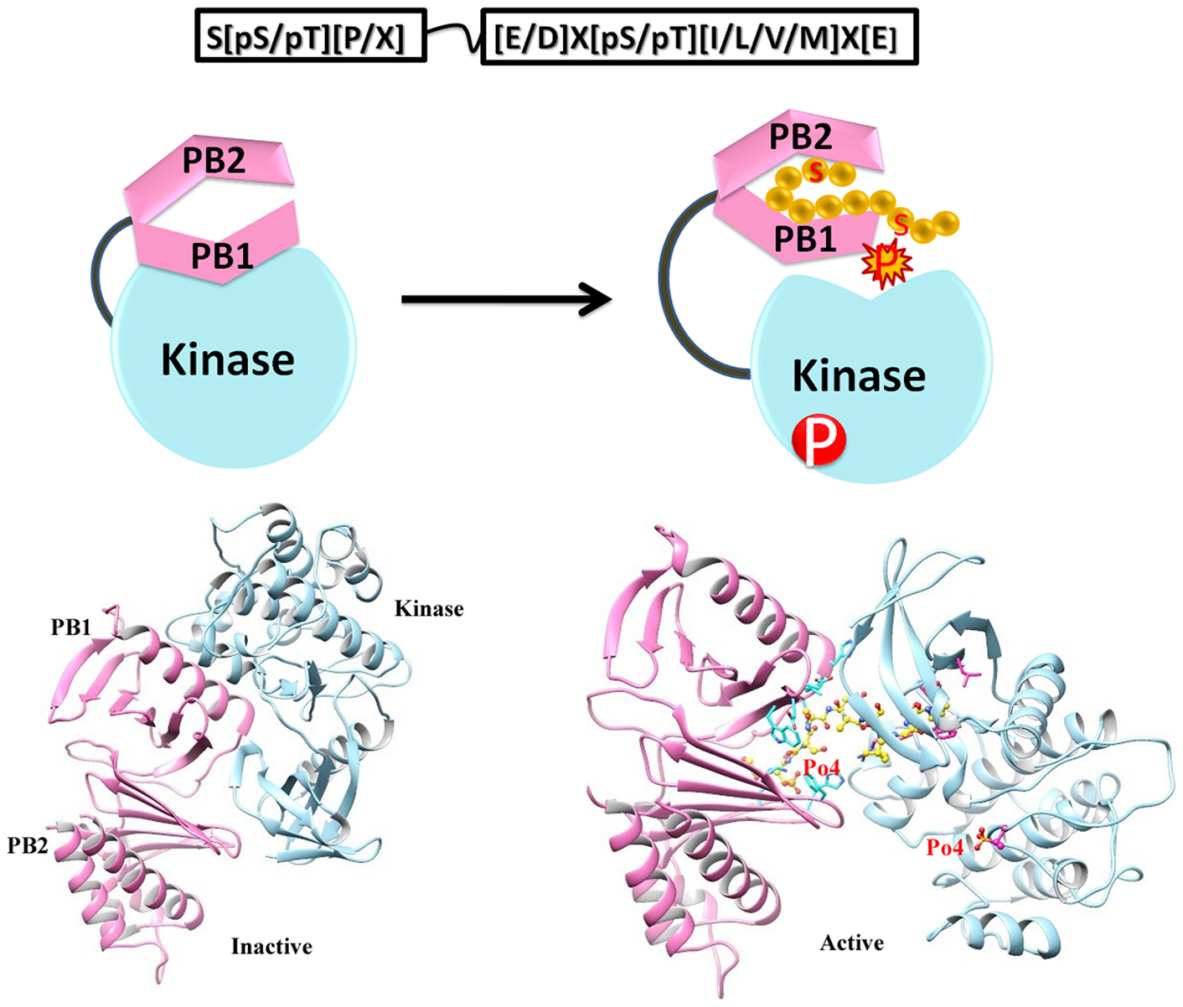
PBD regulated Plk1 kinase activation and substrate phosphorylation. (a) Hypothetical model for the activation of Plk1 proposed by Elia et al., 2003. PBDI and PBDII are shown in pink color, KD in sky blue and bound substrate by yellow balls. (b) Docking analysis data in support of Plk1 activation model. Polo box (pink ribbon) and kinase domains (sky blue ribbon) are tightly bound with each other in an inactive state (left), phosphopeptide (yellow ball and sticks) binding to PBD discharges the KD for phosphorylation (right).

**Figure 8:**
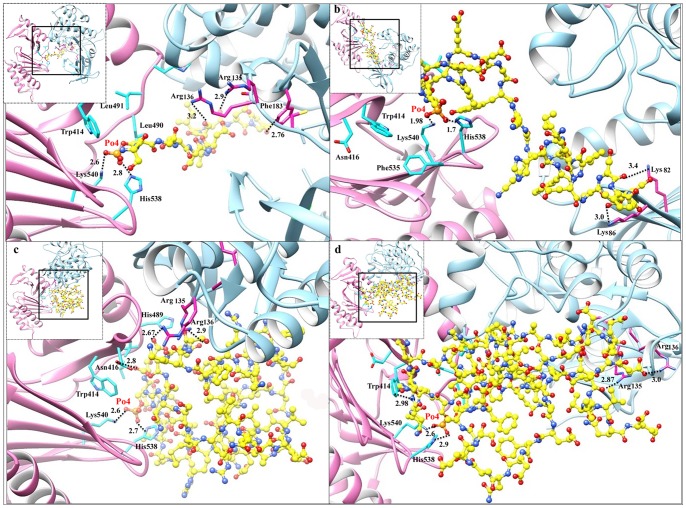
Binding mode and molecular interaction analyses of putative substrates. (I) localization of potential kinase phosphorylation and PBD recognition motif; (a) Plk1-SMARCAD1 complex, (b) Plk1-GSG2 complex, (c) Plk1-KIF23 complex and (d) Plk1-CEP170 complex. KD is shown in blue ribbon with interacting residues in pink sticks, while PBD is shown in pink ribbon and cyan sticks represent interacting residues. Substrate peptides are shown in yellow ball and stick mode and hydrogen bonding is shown by black dotted lines with calculated distances in angstrom.

Through molecular docking analysis, we observed binding of Plk1-PBD to KD through Trp414, Asp416, Tyr417, Asp419, Lys420, Try421, Tyr481, Arg483, Asn484, Tyr485, Ser487, Glu488, Tyr481, Leu490, Leu491 residues, while KD specific residues included Lys61, Lys66, Ser87, Leu88, Glu140, Arg136, Lys143, Thr214, Ans216, Lys178, Glu252, Thr253, Ser254 (Figure 6 and S1). However, subsequent binding of phosphopeptide to PBD resulted in its conformational shift thereby releasing the KD to interact with the nearby phosphorylation motif of target protein (Figure 7). The observed interacting residues of Plk1-PBD (Trp414, Asn416, Tyr485, His489, Phe535, Lue491, Leu492, His538 and Lys540) and KD (Leu59, Gly63, Lys82, Lys86, Cys133, Arg135, Arg136, Ser137 and Phe183) with PBD recognition **(S-[pS/pT]-[P/X])** and the putative phosphorylation **([E/D]X[pS/pT][I/L/V/M]X[E])** motifs, respectively are specified in Figure 8. Based on the scoring functions obtained from the docked conformations (Table 1), in majority of the Plk1 bound substrates, implications of His538 and Lys540 residues (PBD) were detected having strong hydrophilic interactions with the phosphate moiety of phosphopeptide. Thus our docked Plk1 complexes closely resemble the known phosphopeptide bound Plk1 crystal structures (IQ4K, 3P37, 3P2Z and 4E9D) in terms of their binding pattern. Next, to cross validate our results, KFC2 (http://kfc.mitchell-lab.org) and InterProSurf (http://curie.utmb.edu/prosurf.html) servers were used to analyze these docked complexes on the basis of solvent-accessible surface areas, complementarities and surface geometry.

**Table 1:** Multiple scoring functions of selected substrates with Plk1.

	*Binding Energy (kcal/mol)*	*Intermolecular Energy (kcal/mol)*	*ElectrostaticEnergy kcal/mol)*	*vdW + Hbond + desolv Energ (kcal/mol*	*Total Internal Energy (kcal/mol)*	*Torsional Free Energy (kcal/mol)*	*Unbound System*'*s Energy (kcal/mol)*	*Ref RMS(Å)*
**SMARCAD1**	−3.03	−8.00	−2.32	−5.68	−4.60	7.76	−2.90	99.15
**NUP53**	−4.75	−8.68	0.30	−8.98	−5.30	6.68	−2.39	75.23
**NEK5**	−2.24	−8.50	−1.72	−6.78	0.00	6.26	0.00	20.83
**GSG2**	−2.80	−7.68	−1.77	−5.91	0.00	6.68	0.00	13.57
**ZNF791**	−0.93	−8.09	−0.94	−7.4	0.00	7.16	0.00	58.63
**MARK1**	−1.11	−8.27	−1.44	−6.83	0.00	7.16	0.00	137.34
**MARK4**	−1.01	−8.27	−1.00	−6.85	0.00	7.26	0.00	60.78
**HUS1B**	−0.10	−7.00	−1.43	−6.95	0.00	7.30	0.00	65.56
**SIK2**	−2.00	−7.89	−1.56	−7.34	0.00	6.92	0.00	70.67
**TOP3B**	−0.00	−6.68	−1.04	−5.21	0.00	6.23	0.00	47.98
**KIF23**	−5.15	−8.07	−2.34	−8.96	0.16	6.78	−2.03	70.32
**CEP170**	−3.98	−7.99	−1.53	−6.88	0.39	7.05	0.00	56.76
**BRCA2**	−2.35	−8.49	−1.37	−5.87	0.00	6.68	−2.01	24.89

As binding of the phosphopeptide to PBD discharges the KD, PBD serves as an essential molecular mediator to regulate activation and substrate selectivity in time and space. In order to have a deep insight of the conformational switches, docked substrates were ranked on the basis of binding free energy values in parallel to their specificity and affinity assays for Plk1 domains. The accumulated evidence allowed us to establish MD simulation studies in order to track rapid changes in PBD during interaction. Consequently, complexes of four primed phosphorylated targets (SMARCAD1, GSG2, NEK5 and NUP35) with Plk1-PBD were further investigated by MD simulations to monitor their dynamic behavior and stability of interactions.

### Molecular dynamics simulation analysis

**Figure 9:**
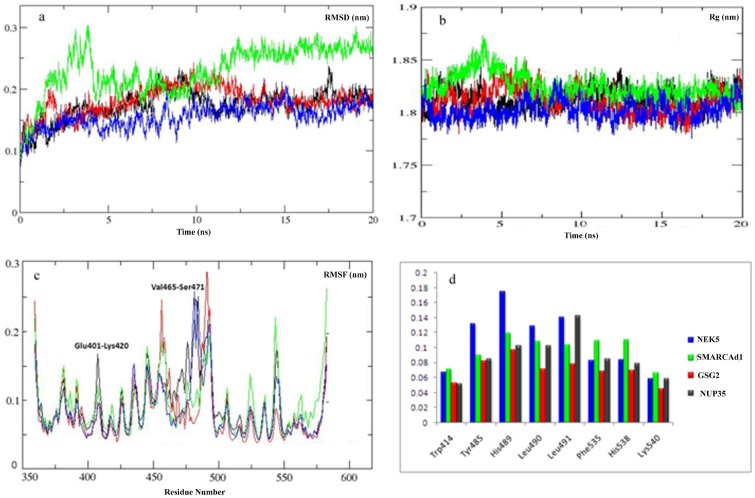
Plots to investigate the stability and fluctuation of MD trajectories for all systems. (a) RMSD plot computed through each system trajectory, (b) Rg plot, (c) RMSF plot (full protein), Plk1-NEK5 complex, (blue) Plk1-GSG2 complex (red), Plk1- SMARCAD1 complex (green), Plk1-NUP35 complex (black), (d) Significant Plk1 residues involved in substrates binding. Overall residues are stable with no more than 1Å fluctuation. Trp414, His538 and Lys540 are much stable in all systems.

The stability of secondary structure elements and conformational changes of simulated complexes were assessed by plotting root mean square deviation (RMSD), root mean square fluctuation (RMSF) and radius of gyration (Rg) values, obtained throughout the trajectory. Backbone RMSD scores observed over a period of 20 ns for GSG2, NEK5 and NUP35 substrates remained stable (2 Å) throughout simulation, however in case of SMARCAD1, RMSD behavior showed a marked increase during 3–4 ns time period, indicating a decrease in stability (Figure 9a). Radius of gyration (Rg) is a simple measure of stability and firmness of the system and tends to change over time due to protein folded-unfolded states [60]. The calculated 2D plots for mean Rg for all the systems were consistent with RMSD profiles (Figure 9b). In agreement to RMSD data, Rg profile for SMARCAD1-Plk1 was elevated during 3–4 ns, indicating a conformational change due to unfolding. These data further validated the stability of kinase-substrate complexes during simulations. Subsequent analysis of root mean square fluctuation (RMSF) by residue indicated more fluctuations (up to 3 Å) in regions proximal to substrate binding residues (400–500 AA) (Figure 9c). These regions exhibiting significantly higher rate of fluctuations included Glu401-Lys420, Ser466-Ser471 and Ile497-Pro500. However, amino acids involved in substrate binding (Trp414, Tyr485, His489, His538, Lys540, Leu490 and Leu491) were stable and exhibited minor fluctuations (less than 1 Å) (Figure 9d).

Next, dynamics of individual simulated complexes were monitored by generating trajectories for 5 ns, 10 ns, 15 ns and 20 ns time intervals to investigate the time-dependent dynamics of individual amino acids for GSG2, NEK5, NUP35 and SMARCAD1 phosphopeptides and their interactions with Plk1. It was observed that these target peptides exhibited a quite stable binding pattern and shared several crucial residues located at the interface of PBDI and PBDII with varying binding affinities (Figure S2).

**Figure 10:**
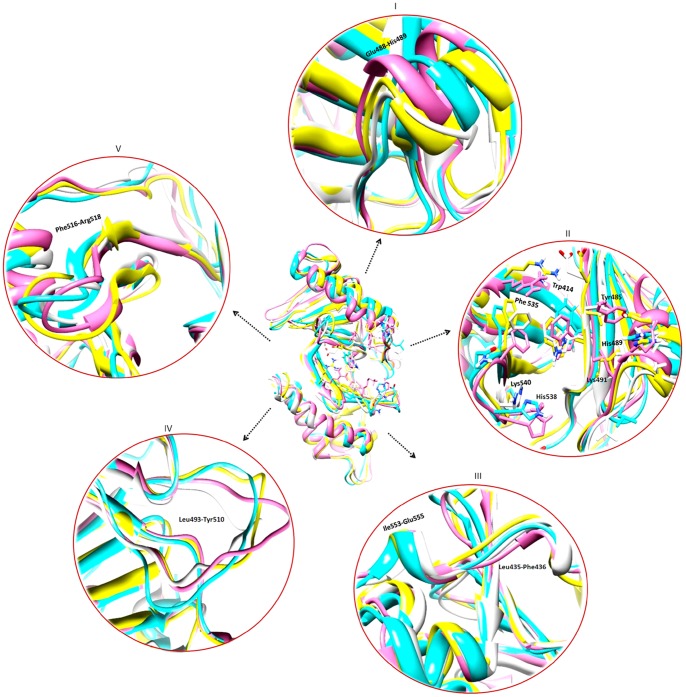
Conformational switches of Plk1 structure upon substrate binding. NEK5 (white), NUP35 (pink), GSG2 (blue) and SMARCAD1 (yellow). (I) Helical region is variable in NEK5, NUP35 and SMARCAD1. (II) Specific binding orientation of active site residues in different complexes. (III) Extension of β-strand at two regions, Ile553-Glu555 in Nup53, GSG2 and SMARCAD1 complexes and Leu435-Phe436 in NEK5, NUP35 complexes. (IV) Changes at linker region between two PBD domains of Plk1. (V) Three residues (Phe516-Arg518) extension of β strand in GSG2 complex.

During MD simulations, significant conformational changes were observed in and around the active site of PBDs, influencing the substrate binding. The first change was observed in Glu488 and His489 residues, a helical region in apo-form; however, upon binding to SMARCAD1 and NUP35 phosphopeptides, the loop is moved up (Figures S3b, S3c and 10-I). This change in helix brought a significant conformational change in His489 residue, pointing towards the binding pocket to interact with substrate. Moreover, in case of GSG2, conformational change in His489 near the substrate binding region aided in interaction (Figure S3a), while in case of NEK5, the ring of His489 tilted away from the substrate, thus rendering this residue restrained and away from the substrate (Figure S3d). Second change induced by peptide binding was extension of two β-strands in the regions of Ile553-Glu555 and Lue435-Phe436 amino acids; however, these extensions did not influence any of the active site residues (Figure 10-III). Third structural change was observed in the region of Glu401-Lys420 in SMARCAD1, NEK5 and NUP35 specific peptide-binding. This region was variable in the apo-form; however, upon substrate binding, a small helix was formed containing three amino acid residues (Pro403-Cys405). In case of NEK5 binding, another helix was formed in the region of Val465-Ser467. In case of GSG2, β-strand was extended by three residues Arg516-Arg518. Moreover, significant changes were observed in the linker region (Figure 10-IV) for all the complexes. These structural changes observed during our analyses in four PBD-phosphopeptide systems clearly depicted specificity of Plk1-PBD binding (Figure 10-II) by allowing flexibility of key residues involved in substrate recognition and localization at the binding pocket. As a result of alteration in Plk1-PBD upon substrate binding, conformational shift may allow Plk1-KD to become free and interact with putative phosphorylation motif of PBD-bound substrate.

## Discussion

Biology of cell-cycle is a powerful area of study with a frequently increasing players and complex regulatory network. Coordinated control of phosphorylation has been extensively considered for tight regulation of cell-cycle. Like many other kinases, Plk1 is a major propeller of cell cycle working beyond the mitosis [61] from DNA replication to cytokinesis [62]. Here we employed amalgamation of strategies to determine Plk1 specific targets involved in cell-cycle and multiple carcinomas, as mapping and chemical modifications of targets in terms of their involvements in precise cell-cycle phase remains very important to address their unique roles in distinct subcellular events.

To date, more than 70 Plk1 substrates have been identified (Table S1) encompassing bulk of spindle proteins; however, identified targets are not enough to broadly explicate the complex job of Plk1 ahead of mitosis. More recently, an extensive assay of Plk1 dependent spindle proteome performed by [63] provided a better ranking of our hits and validated our criteria for the efficient prediction of Plk1 targets (Figure S4). Our identified list of novel Plk1 targets encompasses several proteins implicated in microtubule cytoskeleton organization, spindle dynamics, nuclear division, DNA damage response, DNA repair molecules, chromatin modification, transcription factors and cell cycle regulatory proteins. Subsequent clustering of potential substrates urged the synchronized regulation of Plk1-based targets for particular functions. Notably, more than 40% of potential Plk1-dependent phosphorylation targets identified in our screen were linked to G2/M-phase (Figure 3), concerning with the elevated expression of Plk1 during this phase [64]. Owing to the Plk1 functions in localization and fine tuning of several checkpoint components [61], 10% of our identified targets were localized at the cell-cycle checkpoints. The usefulness of identified hits, grouped on the basis of common functional criteria can be inferred by many examples. For example, E3 ubiquitin-protein ligases (RNF220 and RNF19A) which are involved in controlling the cleavage furrow formation during cytokinesis [65] were predicted as potential Plk1 targets in this study. Similarly, the known Plk1 substrates (CEP170 and KIF23) which contribute in the formation of spindle midzone during cytokinesis [59] were also identified during our screening process. Together, the proposed cytokinesis sub-network (Figure 3) substantiates the role of Plk1 in spatiotemporal regulation of cytokinesis to determine the accurate cleavage plane.

Previously, Plk1 has been reported to collaborate with Aurora A kinase [66] as both are activated during G2-M transition and exhibit specific and overlapping functions by sharing multiple substrate candidates [18] (Figure S5). In our screening data, we also observed several common phosphorylation targets (e.g. HDAC6, BRCA1, member of CEP family, PRKC etc.) of these kinases which conferred their cooperative role in co-regulation and control of key mitotic events. Among the mutual targets, HDAC6 and PRKC meet our stringent filtering criteria set.

Despite the well-defined role of Plk1 in cell-cycle progression, it also acts during DNA synthesis and maintenance of genome stability in DNA replication [67]. Analyzing hits in terms of DNA related functions revealed CHD4 (chromodomain helicase DNA binding protein 4), POLQ (polymerase (DNA directed), theta), TOP3B (topoisomerase (DNA) III beta) and SMARCAD1 (SWI/SNF-related, matrix-associated actin-dependent regulator of chromatin) as potential Plk1 targets which contain consensus PBD binding and phosphorylation signature (Table S1D). These hits signified a novel link of Plk1 in chromatin remodeling; although, their precise mode of action and intricate functional implications remain to be investigated. Another unique hit identified during our screening process was NEK5 (NIMA (never in mitosis gene a)-related kinase 5) which potentiates the role of Plk1 in regulating NIMA-like pathway through cell-cycle dependent manner. NEK5 forms a heterodimer with NEK4 and NEK6 and assists these NEKs in regulating cellular expansion and morphogenesis [68]. NEK5 is strongly regulated during cell-cycle and exhibits analogous function to CDC2 (Cyclin-Dependent Kinase) [69]. More interestingly, in our screen, GSG2 (Germ cell-specific gene 2) was identified as a novel phosphorylation target of Plk1. Having strong expression in testis [70] and function in spermatogenesis [71], GSG2 potentiates additional function of Plk1 in the post-meiotic spermatogenesis. Plk1 has also been reported to regulate members of NPC components including nucleoporins (NUPs) during kinetochore localization [63]. In this study, we identified NUP35, NUP88 and NUP205 as putative Plk1 substrates thereby suggesting the novel regulatory role of NUPs in cell division. Moreover, our data set holds several Zinc finger proteins (zinc finger protein 791 (ZNF791) and pogo transposable element with ZNF domain (POGZ) whose phosphorylation seems to rely on Plk1 binding (Table S1C and D). Collectively, the comprehensive assessment of candidate substrates in precise biological context and understanding of critical regulatory mechanisms linked to each target in cell-cycle dependent manner is obligatory to substantiate whether these identified targets are bona fide substrates of Plk1.

To substantiate our screening results, we selected a few putative targets (known and novel) from each sub network and subjected to structure-based assortment through monitoring PBD mediated interactions and subsequent kinase activation processes. An extensive investigation of the dynamic behavior of four potential substrate peptides (SMARCAD1, GSG2, NEK5 and NUP35) with Plk1 PBD provided a strong indication of substrate specificity. Intriguingly, the key residues (Trp414, Tyr485, His489, His538, Lys540, Leu490 and Leu491) underlying the substrate interactions in our analyses were quite consistent with the earlier studies [14], while the contributions of Asp416, Tyr485, His489 and Phe535 residues implicated in phosphopeptide binding were varying with respect to the individual substrate, which conferred additional insight into PBD binding specificity by changing the binding pocket dynamics. Strikingly, the influence of two basic residues His538 and Lys540 in substrate binding was quite dominating and stable during the MD simulations. Previously, this role of His538 and Lys540 amino acids in phosphopeptide binding has been supported by mutation of these residues into Ala, which resulted in a complete loss of binding [14].

As phospho-dependent ligand recognition by PBD is vital for targeting of Plk1 to specific substrate (processive phosphorylation), it is obvious that diverse conformational changes induced in PBD upon primed phosphopeptide binding facilitate the recruitment of Plk1 KD to the substrate and allow its phosphorylation at the specified motif **([E/D]X[pS/pT][I/L/V/M]X[E])** by relieving the intramolecular inhibitory action (Figure 7). Evidently, it has been well established that Plk1 specific KD and PBD exhibit intramolecular interaction to inhibit the activity of Plk1 and subsequent T-loop phosphorylation at Thr210 results in relieve of this inhibition [72], [73], [74], [75]. Park et al. in 2010 [76] proposed the association between KD and PBD1 to regulate the function of kinase activity. In our assays, we observed dimerization of PBD and KD through interfacial interaction of PBD1 and KD mimicking the mutual inhibition. However, this intramolecular binding was impaired upon PBD contact with phosphopeptide. This conclusion is further supported by the earlier studies where conformational change in the active Plk1 upon phosphopeptide binding disrupted the contact of PBD and KD to stimulate kinase activity [72]. Thus based on these observations, it is concluded that PBD-dependent binding to phosphopeptide and T-loop phosphorylation may cooperatively impair the physical interaction of KD and PBD1 to induce kinase activity. It is possible that in KD, phosphorylation of T-loop specific residues may be regulated through a coordinated interaction with PBD. In agreement to our hypothesis, Thr214 of T-loop which is a known phosphorylation target [77] was identified to make hydrogen bond with Glu488 of PBD. Similarly, in the activation loop of KD, Pro215 and Asn216 residues lying very closely to the phosphorylated Thr210 and Thr214 were also implicated in PBD interactions (Figure 6). Indeed, in our study, the pronounced PBD binding and phosphorylation signature of priming kinases in the identified hits (Table 1) signified their authenticity which is in harmony with the Plk1 directed substrate modification model proposed by Elia et al., 2003 and processive phosphorylation model of Lowery *et al*., 2005, we cannot exclude the possibility of substrate binding to KD without the involvement of priming phosphorylation. Determination of crystal structure of KD bound to PBD and further studies may delineate the diversity of phosphopriming dependent and independent interactions of Plk1 targets.

Collectively, our study reveals several novel functions of Plk1 related to cellular process by extending the inventories of phosphorylation-dependent substrates and data may serve as a valuable resource in the field. Together, exploitation of the novel Plk1 mediated functions specified in this study may open new avenue for cancer therapeutics.

## Conclusion

There have been very few attempts to delineate the potential role of Plk1 in cell-cycle and cancer. Due to an ensemble of interconnecting functions of Plk1 in cell-cycle process and various carcinomas, the urge to describe intricate detail of novel PBD binding targets in conjunction with kinase association is overwhelming. By applying a range of bioinformatics tools and a stringent set of filters, we reliably predicted the cognate partners of Plk1, partly based on the consensus motif information for kinase binding and a common feature of PBD binding. More sophisticated assays revealed the essential residues of PBD, which contributed in substrate binding and allowed it to change the conformation of adjacent KD for phosphorylation. The fact that phosphorylation events deregulate the cell-cycle process during tumorigenesis makes polo-like kinases as key targets for anticancer drugs. Thus, identification of putative Plk1-dependent novel substrates in context to cancer and cell-cycle process will unravel the underlying molecular mechanisms and may prove significant for the assessment of multi-targeted drug candidates.

## Supporting Information

Figure S1 **Molecular interactions between wild type polo box and kinase domains.** PBD (brown) and KD (pink) are tightly bound with each other through hydrogen bonding shown by green dotted lines and hydrophobic interaction represented by comb like structure.
(TIF)

Figure S2 **Simulated complexes at different time scale.** Binding mode and molecular interactions of four simulated complexes including (a) SMARCAD, (b) GSG2, (c) NEK5, and (d) NUP35 at 5 ns, 10 ns, 15 ns and 20 ns, respectively. All systems exhibit binding stability at the indicated time intervals.
(TIF)

Figure S3 **Structural changes observed at the active site of Plk1 for individual system.** (a) GSG2, (b) SMARCAD1, (c) NUP35 and (d) NEK5 complexes. Interacting amino acid residues of Plk1 apoform (light gold) and complex (cyan) are shown in sticks, while bound substrate molecules are shown in pink sticks in all systems. Hydrogen bonds are not shown for clarity.
(TIF)

Figure S4 **Comparative analysis of our data with the reported mass spectrometry data.** Red nodes represent Plk1 substrates specific to each network (present study and Santamaria *et al*., 2010) while overlapping nodes are shown in cyan color.
(TIF)

Figure S5 **Plk1 and Aurora kinase A interactome.** Green node in each network (Plk1 and AURKA) represents interacting partners. Selectable relationship between the target and its interacting partners are shown by lines which are as follow: red lines (down-regulation); green (up-regulation); gray (regulation), purple (co-expression); blue (chemical modification); yellow (physical interaction) and cyan dotted line (predicted protein interactions).
(TIF)

Table S1 **Frequency of Plk1 phosphorylation motif.** **(Table S1-A)** enlists 906 candidates having 80% identity with the Plk1 **[E/D]X[pS/pT][I/L/V/M]X[E]** consensus motif: **(Table S1-B)** enlists proteins clustered on the basis of their involvements in common biological processes, molecular functions and cellular component localization by GO term. Subsequently, statistical significance of hit-rate was computed as the probability *P* to observe the by chance occurrence of each cluster. **(Table S1-C)** enlists 48.7% of the shot listed hits containing PBD binding capabilities on the basis of presence of Plk1 PBD recognition consensus motif [**S-(pT/pS)-(P/X)]**. **(Table S1-D)** enlists final 70 putative Plk1 phosphorylation targets (known and novel) which fulfill the stringent filtering criteria. Known Plk1 targets are highlighted in pink and novel hits are in black font color.
(XLSX)

Table S2 **3D model validation scores of Plk1-KD Plk1 and its substrates.**
(DOCX)

## References

[1] Barr FA, Sillje HH, Nigg EA (2004) Polo-like kinases and the orchestration of cell division. Nat Rev Mol Cell Biol 5: 429–440. 15173822 10.1038/nrm1401

[2] Glover DM (2005) Polo kinase and progression through M phase in Drosophila: a perspective from the spindle poles. Oncogene 24: 230–237. 15640838 10.1038/sj.onc.1208279

[3] van de Weerdt BC, Medema RH (2006) Polo-like kinases: a team in control of the division. Cell Cycle 5: 853–864. 16627997 10.4161/cc.5.8.2692

[4] Llamazares S, Moreira A, Tavares A, Girdham C, Spruce BA, et al (1991) polo encodes a protein kinase homolog required for mitosis in Drosophila. Genes Dev 5: 2153–2165. 1660828 10.1101/gad.5.12a.2153

[5] Sunkel CE, Glover DM (1988) Polo, a mitotic mutant of Drosophila displaying abnormal spindle poles. J Cell Sci 89: 25–38. 3417791 10.1242/jcs.89.1.25

[6] Donaldson MM, Tavares AA, Hagan IM, Nigg EA, Glover DM (2001) The mitotic roles of Polo-like kinase. J Cell Sci 114: 2357–2358. 11559744 10.1242/jcs.114.13.2357

[7] Glover DM, Hagan IM, Tavares AA (1998) Polo-like kinases: a team that plays throughout mitosis. Genes Dev 12: 3777–3787. 9869630 10.1101/gad.12.24.3777

[8] Hamanaka R, Smith MR, O'Connor PM, Maloid S, Mihalic K, et al (1995) Polo-like kinase is a cell cycle-regulated kinase activated during mitosis. J Biol Chem 270: 21086–21091. 7673138 10.1074/jbc.270.36.21086

[9] Nigg EA (1998) Polo-like kinases: positive regulators of cell division from start to finish. Curr Opin Cell Biol 10: 776–783. 9914175 10.1016/s0955-0674(98)80121-x

[10] Archambault V, Glover DM (2009) Polo-like kinases: conservation and divergence in their functions and regulation. Nat Rev Mol Cell Biol 10: 265–275. 19305416 10.1038/nrm2653

[11] Takaki T, Trenz K, Costanzo V, Petronczki M (2008) Polo-like kinase 1 reaches beyond mitosis–cytokinesis, DNA damage response, and development. Curr Opin Cell Biol 20: 650–660. 19000759 10.1016/j.ceb.2008.10.005

[12] Pellegrino R, Calvisi DF, Ladu S, Ehemann V, Staniscia T, et al (2010) Oncogenic and tumor suppressive roles of polo-like kinases in human hepatocellular carcinoma. Hepatology 51: 857–868. 20112253 10.1002/hep.23467

[13] Cheng KY, Lowe ED, Sinclair J, Nigg EA, Johnson LN (2003) The crystal structure of the human polo-like kinase-1 polo box domain and its phospho-peptide complex. Embo J 22: 5757–5768. 14592974 10.1093/emboj/cdg558 PMC275415

[14] Elia AE, Rellos P, Haire LF, Chao JW, Ivins FJ, et al (2003) The molecular basis for phosphodependent substrate targeting and regulation of Plks by the Polo-box domain. Cell 115: 83–95. 14532005 10.1016/s0092-8674(03)00725-6

[15] Kang YH, Park JE, Yu LR, Soung NK, Yun SM, et al (2006) Self-regulated Plk1 recruitment to kinetochores by the Plk1-PBIP1 interaction is critical for proper chromosome segregation. Mol Cell 24: 409–422. 17081991 10.1016/j.molcel.2006.10.016

[16] Lowery DM, Clauser KR, Hjerrild M, Lim D, Alexander J, et al (2007) Proteomic screen defines the Polo-box domain interactome and identifies Rock2 as a Plk1 substrate. Embo J 26: 2262–2273. 17446864 10.1038/sj.emboj.7601683 PMC1864981

[17] Neef R, Preisinger C, Sutcliffe J, Kopajtich R, Nigg EA, et al (2003) Phosphorylation of mitotic kinesin-like protein 2 by polo-like kinase 1 is required for cytokinesis. J Cell Biol 162: 863–875. 12939256 10.1083/jcb.200306009 PMC2172827

[18] Archambault V, Glover DM (2008) Yeast Polo-like kinase substrates are nailed with the right tools. Genome Biol 9: 203. 18254925 10.1186/gb-2008-9-1-203 PMC2395232

[19] Snead JL, Sullivan M, Lowery DM, Cohen MS, Zhang C, et al (2007) A coupled chemical-genetic and bioinformatic approach to Polo-like kinase pathway exploration. Chem Biol 14: 1261–1272. 18022565 10.1016/j.chembiol.2007.09.011 PMC2215327

[20] Toyoshima-Morimoto F, Taniguchi E, Shinya N, Iwamatsu A, Nishida E (2001) Polo-like kinase 1 phosphorylates cyclin B1 and targets it to the nucleus during prophase. Nature 410: 215–220. 11242082 10.1038/35065617

[21] Lin HR, Ting NS, Qin J, Lee WH (2003) M phase-specific phosphorylation of BRCA2 by Polo-like kinase 1 correlates with the dissociation of the BRCA2-P/CAF complex. J Biol Chem 278: 35979–35987. 12815053 10.1074/jbc.M210659200

[22] Casenghi M, Meraldi P, Weinhart U, Duncan PI, Korner R, et al (2003) Polo-like kinase 1 regulates Nlp, a centrosome protein involved in microtubule nucleation. Dev Cell 5): 113–125. 12852856 10.1016/s1534-5807(03)00193-x

[23] Yarm FR (2002) Plk phosphorylation regulates the microtubule-stabilizing protein TCTP. Mol Cell Biol 22: 6209–6221. 12167714 10.1128/MCB.22.17.6209-6221.2002 PMC134017

[24] Lee KS, Yuan YL, Kuriyama R, Erikson RL (1995) Plk is an M-phase-specific protein kinase and interacts with a kinesin-like protein, CHO1/MKLP-1. Mol Cell Biol 15: 7143–7151. 8524282 10.1128/mcb.15.12.7143 PMC230970

[25] Liu X, Zhou T, Kuriyama R, Erikson RL (2004) Molecular interactions of Polo-like-kinase 1 with the mitotic kinesin-like protein CHO1/MKLP-1. J Cell Sci 117: 3233–3246. 15199097 10.1242/jcs.01173

[26] Nakajima H, Toyoshima-Morimoto F, Taniguchi E, Nishida E (2003) Identification of a consensus motif for Plk (Polo-like kinase) phosphorylation reveals Myt1 as a Plk1 substrate. J Biol Chem 278: 25277–25280. 12738781 10.1074/jbc.C300126200

[27] Jackman M, Lindon C, Nigg EA, Pines J (2003) Active cyclin B1-Cdk1 first appears on centrosomes in prophase. Nat Cell Biol 5: 143–148. 12524548 10.1038/ncb918

[28] Zhou T, Aumais JP, Liu X, Yu-Lee LY, Erikson RL (2003) A role for Plk1 phosphorylation of NudC in cytokinesis. Dev Cell 5: 127–138. 12852857 10.1016/s1534-5807(03)00186-2

[29] Lin CY, Madsen ML, Yarm FR, Jang YJ, Liu X, et al (2000) Peripheral Golgi protein GRASP65 is a target of mitotic polo-like kinase (Plk) and Cdc2. Proc Natl Acad Sci U S A 97: 12589–12594. 11050165 10.1073/pnas.220423497 PMC18808

[30] Sutterlin C, Lin CY, Feng Y, Ferris DK, Erikson RL, et al (2001) Polo-like kinase is required for the fragmentation of pericentriolar Golgi stacks during mitosis. Proc Natl Acad Sci U S A 98: 9128–9132. 11447294 10.1073/pnas.161283998 PMC55384

[31] Sakchaisri K, Asano S, Yu LR, Shulewitz MJ, Park CJ, et al (2004) Coupling morphogenesis to mitotic entry. Proc Natl Acad Sci U S A 101: 4124–4129. 15037762 10.1073/pnas.0400641101 PMC384705

[32] Watanabe N, Arai H, Nishihara Y, Taniguchi M, Watanabe N, et al (2004) M-phase kinases induce phospho-dependent ubiquitination of somatic Wee1 by SCFbeta-TrCP. Proc Natl Acad Sci U S A 101: 4419–4424. 15070733 10.1073/pnas.0307700101 PMC384762

[33] Sharan R, Ulitsky I, Shamir R (2007) Network-based prediction of protein function. Mol Syst Biol 3: 88. 17353930 10.1038/msb4100129 PMC1847944

[34] Altschul SF, Madden TL, Schaffer AA, Zhang J, Zhang Z, et al (1997) Gapped BLAST and PSI-BLAST: a new generation of protein database search programs. Nucleic Acids Res 25: 3389–3402. 9254694 10.1093/nar/25.17.3389 PMC146917

[35] Ashburner M, Ball CA, Blake JA, Botstein D, Butler H, et al (2000) Gene ontology: tool for the unification of biology. The Gene Ontology Consortium. Nat Genet 25: 25–29. 10802651 10.1038/75556 PMC3037419

[36] Milenkovic T, Przulj N (2008) Uncovering biological network function via graphlet degree signatures. Cancer Inform 6: 257–273. 19259413 PMC2623288

[37] Blom N, Sicheritz-Ponten T, Gupta R, Gammeltoft S, Brunak S (2004) Prediction of post-translational glycosylation and phosphorylation of proteins from the amino acid sequence. Proteomics 4: 1633–1649. 15174133 10.1002/pmic.200300771

[38] Zhou FF, Xue Y, Chen GL, Yao X (2004) GPS: a novel group-based phosphorylation predicting and scoring method. Biochem Biophys Res Commun 325: 1443–1448. 15555589 10.1016/j.bbrc.2004.11.001

[39] Diella F, Cameron S, Gemund C, Linding R, Via A, et al (2004) Phospho.ELM: a database of experimentally verified phosphorylation sites in eukaryotic proteins. BMC Bioinformatics 5: 79. 15212693 10.1186/1471-2105-5-79 PMC449700

[40] Hubbard TJ, Aken BL, Ayling S, Ballester B, Beal K, et al (2009) Ensembl 2009. Nucleic Acids Res 37: D690–697. 19033362 10.1093/nar/gkn828 PMC2686571

[41] Sali A, Blundell TL (1993) Comparative protein modelling by satisfaction of spatial restraints. J Mol Biol 234: 779–815. 8254673 10.1006/jmbi.1993.1626

[42] Zhang Y (2008) I-TASSER server for protein 3D structure prediction. BMC Bioinformatics 9: 40. 18215316 10.1186/1471-2105-9-40 PMC2245901

[43] Wu S, Zhang Y (2008) MUSTER: Improving protein sequence profile-profile alignments by using multiple sources of structure information. Proteins 72: 547–556. 18247410 10.1002/prot.21945 PMC2666101

[44] Colovos C, Yeates TO (1993) Verification of protein structures: patterns of nonbonded atomic interactions. Protein Sci 2: 1511–1519. 8401235 10.1002/pro.5560020916 PMC2142462

[45] Eisenberg D, Luthy R, Bowie JU (1997) VERIFY3D: assessment of protein models with three-dimensional profiles. Methods Enzymol 277: 396–404. 9379925 10.1016/s0076-6879(97)77022-8

[46] Vriend G (1990) WHAT IF: A molecular modeling and drug design program. J Mol Graph 8: 52–56. 2268628 10.1016/0263-7855(90)80070-v

[47] Emsley P, Lohkamp B, Scott WG, Cowtan K (2010) Features and development of Coot. Acta Crystallogr D 66: 486–501. 20383002 10.1107/S0907444910007493 PMC2852313

[48] Meng EC, Pettersen EF, Couch GS, Huang CC, Ferrin TE (2006) Tools for integrated sequence-structure analysis with UCSF Chimera. BMC Bioinformatics 7: 339. 16836757 10.1186/1471-2105-7-339 PMC1570152

[49] Morris GM, Huey R, Lindstrom W, Sanner MF, Belew RK, et al (2009) AutoDock4 and AutoDockTools4: Automated docking with selective receptor flexibility. J Comput Chem 30: 2785–2791. 19399780 10.1002/jcc.21256 PMC2760638

[50] Dominguez C, Boelens R, Bonvin AM (2003) HADDOCK: a protein-protein docking approach based on biochemical or biophysical information. J Am Chem Soc 125: 1731–1737. 12580598 10.1021/ja026939x

[51] Torchala M, Moal IH, Chaleil RA, Fernandez-Recio J, Bates PA (2013) SwarmDock: a server for flexible protein-protein docking. Bioinformatics 29: 807–809. 23343604 10.1093/bioinformatics/btt038

[52] Wallace AC, Laskowski RA, Thornton JM (1995) LIGPLOT: a program to generate schematic diagrams of protein-ligand interactions. Protein Eng 8: 127–134. 7630882 10.1093/protein/8.2.127

[53] Duan Y, Wu C, Chowdhury S, Lee MC, Xiong G, et al (2003) A point-charge force field for molecular mechanics simulations of proteins based on condensed-phase quantum mechanical calculations. J Comput Chem 24: 1999–2012. 14531054 10.1002/jcc.10349

[54] Zlenko DV (2012) [Diffusion factor calculation for TIP4P model of water]. Biofizika 57: 197–204. 22594273

[55] Humphrey W, Dalke A, Schulten K (1996) VMD: visual molecular dynamics. J Mol Graph 14: 33–38, 27–38. 10.1016/0263-7855(96)00018-5 8744570

[56] Larkin MA, Blackshields G, Brown NP, Chenna R, McGettigan PA, et al (2007) Clustal W and Clustal X version 2.0. Bioinformatics 23: 2947–2948. 17846036 10.1093/bioinformatics/btm404

[57] McGuffin LJ, Bryson K, Jones DT (2000) The PSIPRED protein structure prediction server. Bioinformatics 16: 404–405. 10869041 10.1093/bioinformatics/16.4.404

[58] Neduva V, Linding R, Su-Angrand I, Stark A, de Masi F, et al (2005) Systematic discovery of new recognition peptides mediating protein interaction networks. PLoS Biol 3: e405. 16279839 10.1371/journal.pbio.0030405 PMC1283537

[59] Guarguaglini G, Duncan PI, Stierhof YD, Holmstrom T, Duensing S, et al (2005) The forkhead-associated domain protein Cep170 interacts with Polo-like kinase 1 and serves as a marker for mature centrioles. Mol Biol Cell 16: 1095–1107. 15616186 10.1091/mbc.E04-10-0939 PMC551476

[60] Ivankov DN, Bogatyreva NS, Lobanov MY, Galzitskaya OV (2009) Coupling between properties of the protein shape and the rate of protein folding. PLoS One 4: e6476. 19649298 10.1371/journal.pone.0006476 PMC2714458

[61] Liu XS, Song B, Liu X (2010) The substrates of Plk1, beyond the functions in mitosis. Protein Cell 1: 999–1010. 21153517 10.1007/s13238-010-0131-x PMC4875153

[62] Song B, Liu XS, Liu X (2012) Polo-like kinase 1 (Plk1): an Unexpected Player in DNA Replication. Cell Div 7: 3. 22309699 10.1186/1747-1028-7-3 PMC3359159

[63] Santamaria A, Wang B, Elowe S, Malik R, Zhang F, et al (2010) The Plk1-dependent phosphoproteome of the early mitotic spindle. Mol Cell Proteomics 10: M110.004457. 10.1074/mcp.M110.004457 PMC3013462 20860994

[64] Rizkallah R, Alexander KE, Kassardjian A, Luscher B, Hurt MM (2011) The transcription factor YY1 is a substrate for Polo-like kinase 1 at the G2/M transition of the cell cycle. PLoS One 6: e15928. 21253604 10.1371/journal.pone.0015928 PMC3017090

[65] Brennan IM, Peters U, Kapoor TM, Straight AF (2007) Polo-like kinase controls vertebrate spindle elongation and cytokinesis. PLoS One 2: e409. 17476331 10.1371/journal.pone.0000409 PMC1853238

[66] Macurek L, Lindqvist A, Lim D, Lampson MA, Klompmaker R, et al (2008) Polo-like kinase-1 is activated by aurora A to promote checkpoint recovery. Nature 455: 119–123. 18615013 10.1038/nature07185

[67] Yim H, Erikson RL (2009) Polo-like kinase 1 depletion induces DNA damage in early S prior to caspase activation. Mol Cell Biol 29: 2609–2621. 19289504 10.1128/MCB.01277-08 PMC2682042

[68] Motose H, Hamada T, Yoshimoto K, Murata T, Hasebe M, et al (2011) NIMA-related kinases 6, 4, and 5 interact with each other to regulate microtubule organization during epidermal cell expansion in Arabidopsis thaliana. Plant J 67: 993–1005. 21605211 10.1111/j.1365-313X.2011.04652.x

[69] Higgins JM (2010) Haspin: a newly discovered regulator of mitotic chromosome behavior. Chromosoma 119: 137–147. 19997740 10.1007/s00412-009-0250-4 PMC2839057

[70] Lu KP, Hunter T (1995) The NIMA kinase: a mitotic regulator in Aspergillus nidulans and vertebrate cells. Prog Cell Cycle Res 1: 187–205. 9552363 10.1007/978-1-4615-1809-9_15

[71] Wykes SM, Krawetz SA (2003) The structural organization of sperm chromatin. J Biol Chem 278: 29471–29477. 12775710 10.1074/jbc.M304545200

[72] Jang YJ, Lin CY, Ma S, Erikson RL (2002) Functional studies on the role of the C-terminal domain of mammalian polo-like kinase. Proc Natl Acad Sci USA 99: 1984–1989. 11854496 10.1073/pnas.042689299 PMC122306

[73] Jang YJ, Ma S, Terada Y, Erikson RL (2002) Phosphorylation of threonine 210 and the role of serine 137 in the regulation of mammalian polo-like kinase. J Biol Chem 277: 44115–44120. 12207013 10.1074/jbc.M202172200

[74] Lowery DM, Lim D, Yaffe MB (2005) Structure and function of Polo-like kinases. Oncogene 24: 248–259. 15640840 10.1038/sj.onc.1208280

[75] Seki A, Coppinger JA, Jang CY, Yates JR, Fang G (2008) Bora and the kinase Aurora a cooperatively activate the kinase Plk1 and control mitotic entry. Science 320: 1655–1658. 18566290 10.1126/science.1157425 PMC2834883

[76] Park JE, Soung NK, Johmura Y, Kang YH, Liao C, et al (2010) Polo-box domain: a versatile mediator of polo-like kinase function. Cell Mol Life Sci. 67: 1957–1970. 10.1007/s00018-010-0279-9 PMC2877763 20148280

[77] Dulla K, Daub H, Hornberger R, Nigg EA, Körner R (2010) Quantitative site-specific phosphorylation dynamics of human protein kinases during mitotic progression. Mol Cell Proteomics 9: 1167–1181. 20097925 10.1074/mcp.M900335-MCP200 PMC2877978

