# Supplementary figures and images for: Identification of Potential Plk1 Targets in a Cell-Cycle Specific Proteome through Structural Dynamics of Kinase and Polo Box-Mediated Interactions
Source: PLoS One. 2013 Aug 15;8(8):e70843. doi: 10.1371/journal.pone.0070843 (PMC3744538; doi:10.1371/journal.pone.0070843)

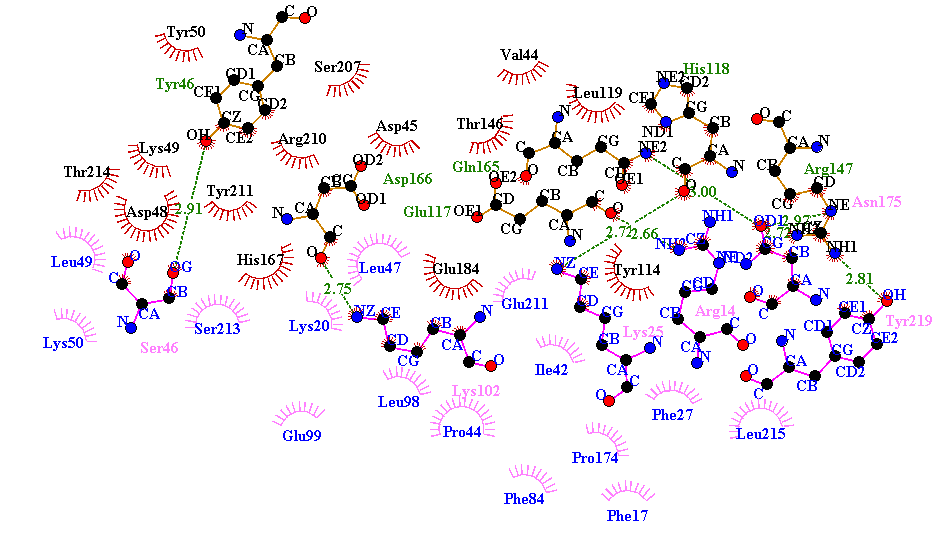

Supplement: Figure S1 — Molecular interactions between wild type polo box and kinase domains. PBD (brown) and KD (pink) are tightly bound with each other through hydrogen bonding shown by green dotted lines and hydrophobic interaction represented by comb like structure. (TIF) [file pone.0070843.s001.tif]

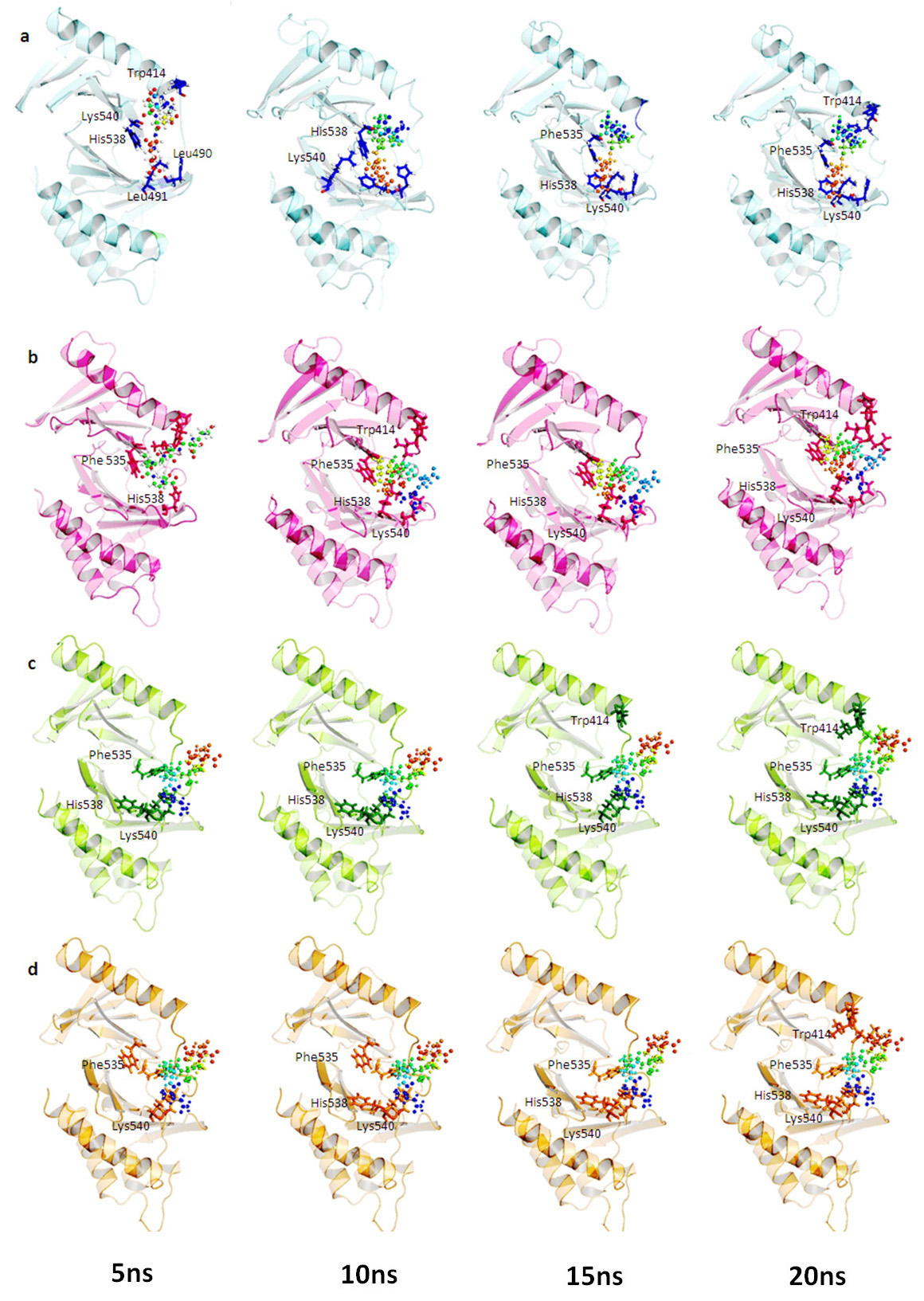

Supplement: Figure S2 — Simulated complexes at different time scale. Binding mode and molecular interactions of four simulated complexes including (a) SMARCAD, (b) GSG2, (c) NEK5, and (d) NUP35 at 5 ns, 10 ns, 15 ns and 20 ns, respectively. All systems exhibit binding stability at the indicated time intervals. (TIF) [file pone.0070843.s002.tif]

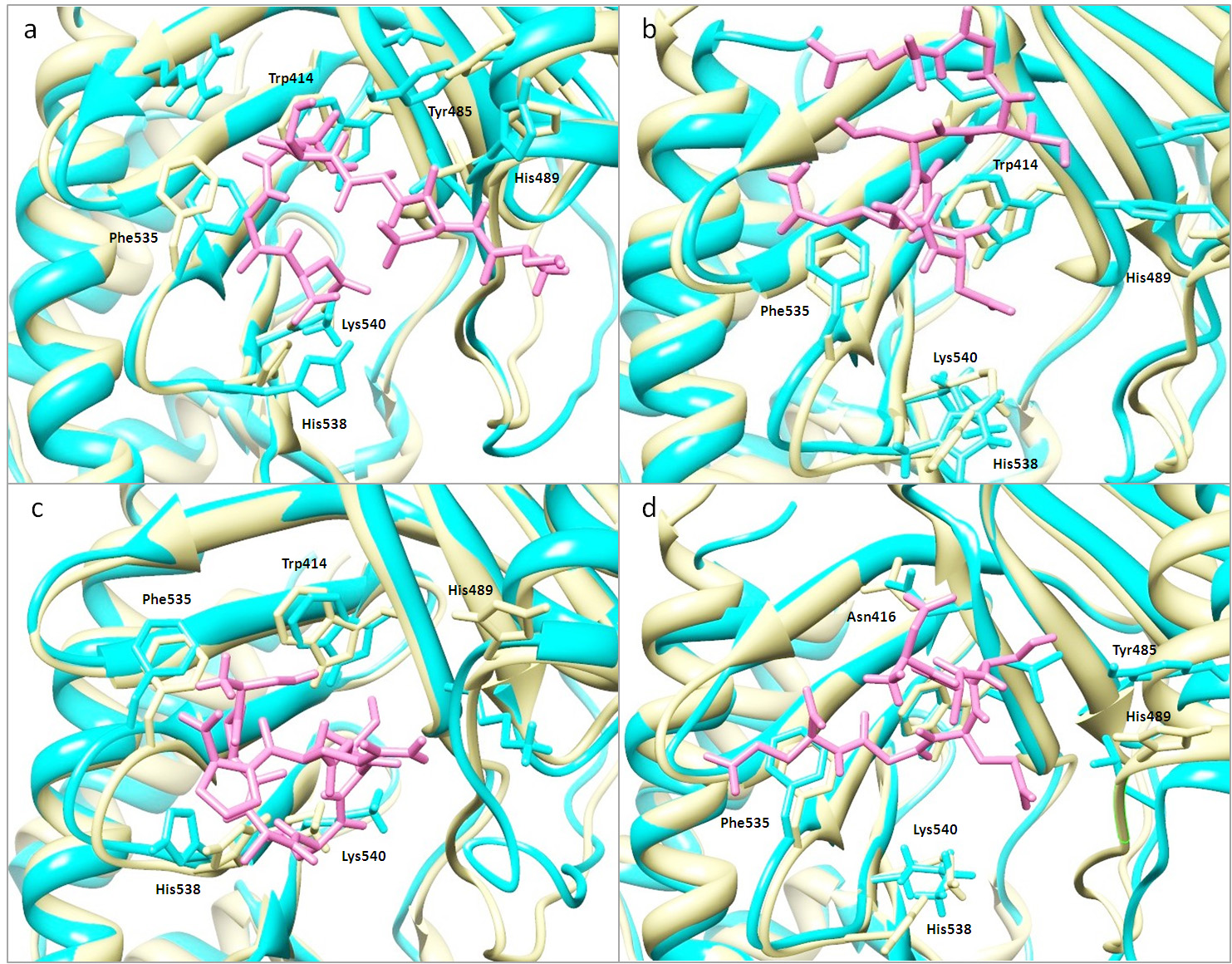

Supplement: Figure S3 — Structural changes observed at the active site of Plk1 for individual system. (a) GSG2, (b) SMARCAD1, (c) NUP35 and (d) NEK5 complexes. Interacting amino acid residues of Plk1 apoform (light gold) and complex (cyan) are shown in sticks, while bound substrate molecules are shown in pink sticks in all systems. Hydrogen bonds are not shown for clarity. (TIF) [file pone.0070843.s003.tif]

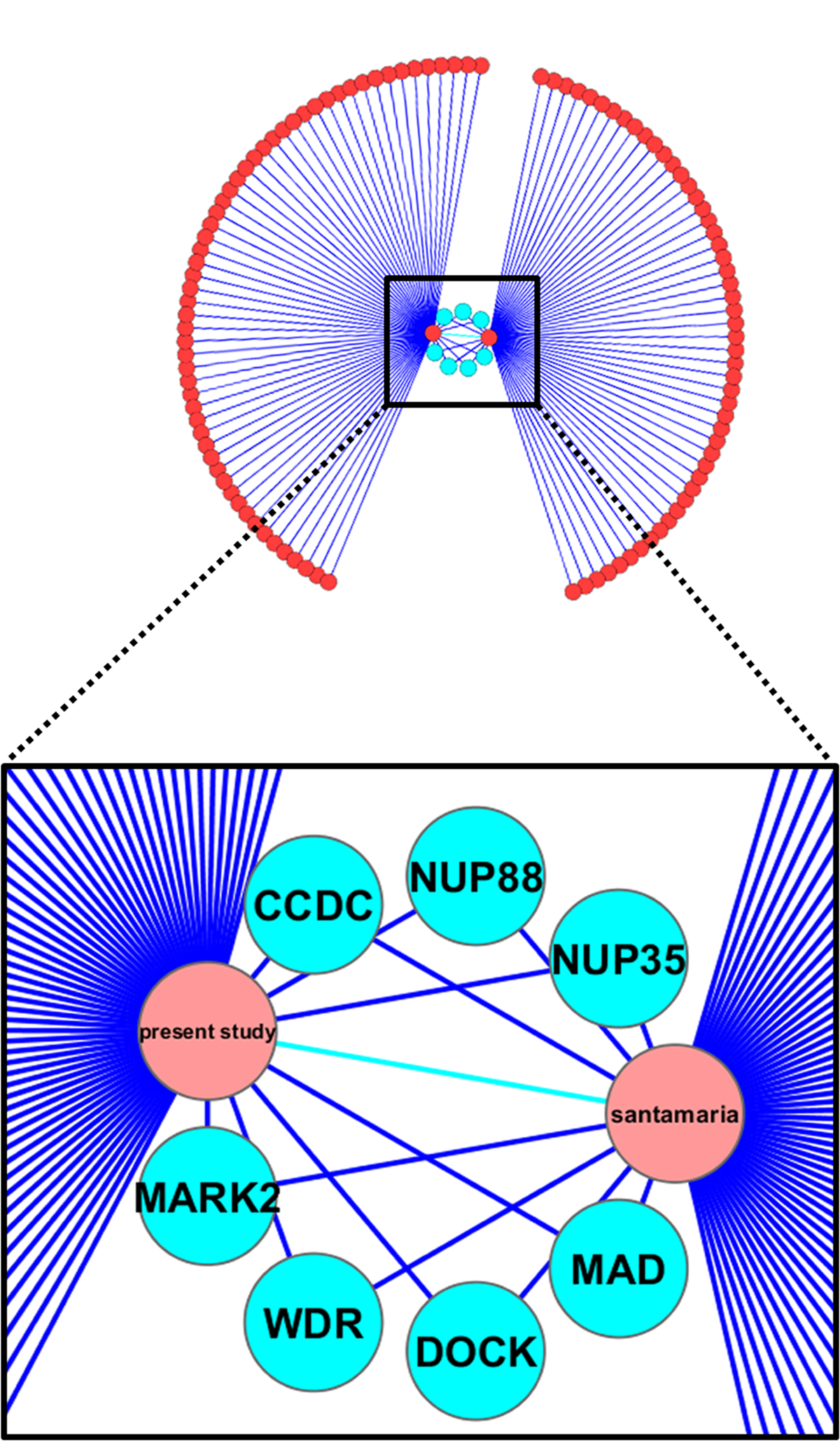

Supplement: Figure S4 — Comparative analysis of our data with the reported mass spectrometry data. Red nodes represent Plk1 substrates specific to each network (present study and Santamaria et al., 2010) while overlapping nodes are shown in cyan color. (TIF) [file pone.0070843.s004.tif]

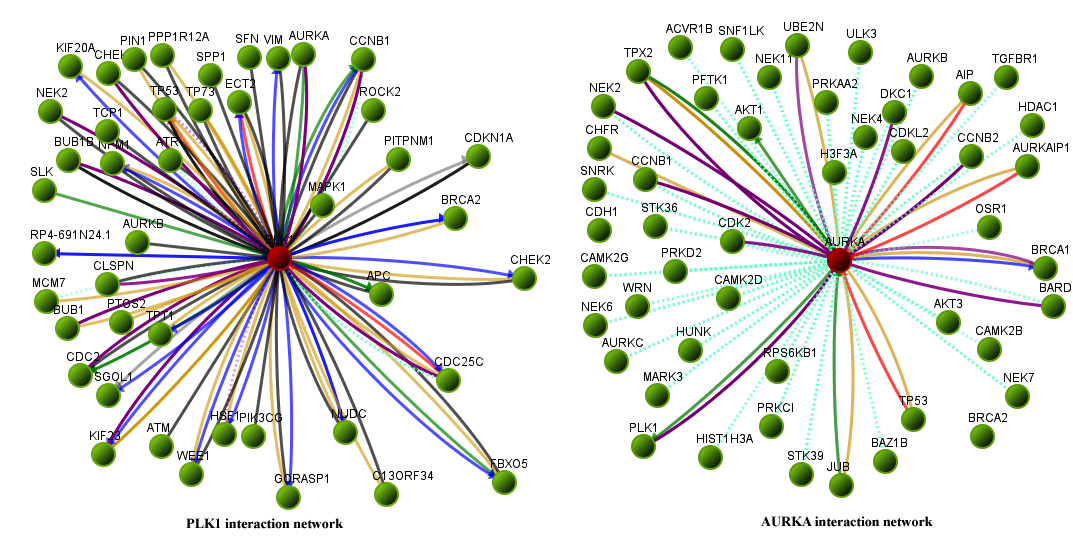

Supplement: Figure S5 — Plk1 and Aurora kinase A interactome. Green node in each network (Plk1 and AURKA) represents interacting partners. Selectable relationship between the target and its interacting partners are shown by lines which are as follow: red lines (down-regulation); green (up-regulation); gray (regulation), purple (co-expression); blue (chemical modification); yellow (physical interaction) and cyan dotted line (predicted protein interactions). (TIF) [file pone.0070843.s005.tif]
