# Supplementary material for: Identification of Potential Plk1 Targets in a Cell-Cycle Specific Proteome through Structural Dynamics of Kinase and Polo Box-Mediated Interactions
Source: PLoS One. 2013 Aug 15;8(8):e70843. doi: 10.1371/journal.pone.0070843 (PMC3744538; doi:10.1371/journal.pone.0070843)
Supplement: Table S2 — 3D model validation scores of Plk1-KD Plk1 and its substrates. (DOCX) [file pone.0070843.s007.docx]

| **Protein name** | **Ramachandran** | **Porcheck** | | **Verify 3D** | **Errat** |
| --- | --- | --- | --- | --- | --- |
| Plk1-KD | 94.03% | 93.96% | | 93.04% | 92.34% |
| Smarcad1 | 99.00% | 96.00% | | 95.09% | 97.00% |
| Nup53 | 98.08% | 96.05% | | 95.06% | 96.08% |
| Nek5 | 97.09% | 95.02% | | 95.00% | 94.00% |
| Gsg2 | 93.04% | 92.07% | | 94.03% | 89.02% |
| Znf791 | 97.00% | 94.09% | | 94.05% | 94.00% |
| Mark1 | 95.00% | 94.00% | | 93.07% | 93.05% |
| Mark4 | 98.00% | 97.01% | | 96.04% | 96.00% |
| Hus1b | 94.08% | 93.7% | | 93.05% | 91.03% |
| Sik2 | 95.00% | 94.08% | | 93.02% | 92.00% |
| Top3b | 98.07% | 97.00% | | 96.03% | 95.04% |
| Kif23 | 98.99% | 97.05% | | 96.06% | 97.88% |
| Cep170 | 97.34% | 96.55% | | 95.66% | 96.23% |
| Brca2 | 97.87% | 96.98% | | 95.77% | 96.86% |
|  | | |  |  |  |

**Table S2**: **3D model validation scores of Plk1-KD and its substrates**
